# Use of palmitoylethanolamide in carpal tunnel syndrome: a prospective randomized study 

**DOI:** 10.1007/s10195-017-0453-z

**Published:** 2017-03-15

**Authors:** Jordi Faig-Martí, Adriana Martínez-Catassús

**Affiliations:** Hospital Sant Rafael, Pg. Vall d’Hebron 107-117, 08035 Barcelona, Catalonia Spain

**Keywords:** Palmitoylethanolamide, Carpal tunnel syndrome, Randomized control trial

## Abstract

**Background:**

Palmitoylethanolamide (PEA) is an endogenous fatty acid amide that has shown anti-inflammatory activity and neuroprotection and has been used for the treatment of compressive syndromes. The aim of this study is to investigate the clinical and electrophysiological effects of conservative treatment with PEA in low to moderate carpal tunnel syndrome (CTS).

**Materials and methods:**

A prospective double-blinded randomized study was performed on 61 patients with a clinical and electrophysiologically confirmed diagnosis of low and moderate CTS. The patients were randomly assigned to two groups. Group *N* was given 300 mg of PEA twice a day over 60 days and Group *P* received a placebo with exactly the same appearance every 12 h for the same period. CTS was evaluated before and after treatment through clinical findings, Boston Carpal Tunnel Questionnaire, visual analog scale (VAS) and electrophysiological data. The results were evaluated with Student’s *t* test and chi-squared test.

**Results:**

No differences were observed in either group compared to the initial status regarding Durkan’s test, Phalen’s test, VAS and electrophysiological data after treatment. The Boston Questionnaire showed better results in both groups, with an improvement in only the symptom severity scale (SSS; *p* = 0.002809) for group *P* and improvement in the functional status scale (FSS; *p* = 0.03334) and SSS (*p* = 0.005) for group *N*.

**Conclusions:**

The results of this study suggest that treatment of CTS with PEA at a dose of 600 mg/day is not associated with an improvement of any clinical and electrophysiological parameters. However, we observed an improvement in the FSS in the Boston Questionnaire after treatment with PEA. Together with the results of other studies, we conclude that further studies of PEA in CTS at higher doses are necessary.

**Level of evidence:**

Level I of evidence according to ‘The Oxford 2011 Level of Evidence’.

## Introduction 

Carpal tunnel syndrome (CTS) is the most common compression neuropathy, with a population prevalence of 4%, which is higher in women (3–5.6%) than in men (0.6–2.8%) [1, 2]. It is due to the compression of the median nerve in the passage through the carpal tunnel in the wrist. It is diagnosed with clinical criteria and electrophysiological studies, which are objective and quantitative, and have been recognized to be highly sensitive and specific for the assessment of nerve function in CTS patients [2].

CTS therapies can be conservative or surgical, but it remains unclear to physicians when it needs treatment, if the severity is low, and how to make a decision regarding surgical treatment [2]. Surgical treatment is indicated in severe cases. In general, conservative management of CTS includes avoiding all provoking factors, correcting potential medical illnesses that can determine CTS (hypothyroidism or diabetes), use of wrist splints, non-steroidal anti-inflammatory drugs, corticosteroids, vitamin B12 and, recently, the use of palmitoylethanolamide (PEA) has been introduced [1].

PEA, first described in 1957 [3], is an endogenous fatty acid amide that has shown anti-inflammatory activity in different experimental models [1, 4–7], as well as analgesia in acute and inflammatory pain [1, 8, 9] and neuroprotection [1, 10–13]. Probably due to the fact that PEA is an endogenous modulator as well as a compound in food, such as eggs and milk, no serious side-effects or any drug interactions have been reported [3, 10].

Clinical trials have evaluated the efficacy and safety of PEA in nerve compression, especially in sciatic pain and pain due to carpal tunnel syndrome [3]. However, it can be useful in other peripheral neuropathies such as chemotherapy-induced peripheral neuropathy, diabetic neuropathy, osteoarthritis, failed back surgery syndrome, dental pain, neuropathic pain in strokes, multiple sclerosis, chronic pelvic pain, post-herpetic neuralgia and vaginal pain [10]. The published literature on the clinical use of PEA is scarce but some authors have reported good results with PEA in the treatment of several peripheral neurological conditions. Randomized controlled studies are needed to clarify the role of PEA in the treatment of such conditions.

The aim of the present study is to prove the potential efficacy and safety of PEA in the treatment of CTS of low and moderate severity by analyzing clinical and electrophysiological changes.

## Materials and methods

A total of 68 patients with a clinical and electrophysiologically confirmed diagnosis of low to moderate carpal tunnel syndrome were recruited for this study and randomly assigned to two groups between February 2011 and September 2014. The study was previously approved by the Ethics Committee of our Institution. Patients with a history of upper extremity surgery, currently taking steroids, using night splinting or with food allergies were excluded. Inclusion criteria were patients aged between 18 and 75 years who were suffering from low or moderate CTS for at least 3 months and who agreed to participate in the study after providing signed informed consent. Group *N* was given 300 mg of PEA twice a day over 60 days. Group *P* was given a placebo with exactly the same appearance twice a day for the same period. After treatment, electroneurography (ENG) was performed and the patient was clinically evaluated and any complications that presented due to medication were recorded. Two patients did not complete the treatment (one in each group), for causes unrelated to the medication, and five more did not receive a follow-up ENG due to refusal or losing the report. The remaining 61 patients included 18 females and 12 males in group *N*, and 19 females and 12 males in group *P*. In 36 cases, the right hand was affected and in 25 cases the left hand was affected. Associated pathologies included fibromyalgia in 3 cases in group *N*.

The severity of symptoms and functional impairment of the patients were assessed using a Spanish translation of Levine’s questionnaire [14, 15] before and after treatment. This tool has good validity, reliability and responsiveness [16] and is recommended as an assessment instrument for research in CTS [17]. The symptom severity scale (SSS) refers to the first 11 questions and the functional status scale (FSS) refers to the last 8 questions of this questionnaire. A clinical examination including Durkan’s test [18] and Phalen’s test [19] was performed at these two time points, and the presence of paresthesia was also recorded. A second ENG was also carried out to evaluate any changes in electrophysiological data.

The results were analyzed using the R statistical package [20] with a *p* value of <0.05. The two groups were compared for differences in the presence of males and females, the average age, weight, body mass index (BMI) and pre-treatment data including Durkan’s test, Phalen’s test, pain in a visual analog scale (VAS), Levine’s questionnaire, and differences in electrophysiological data. The *t* test was used for data that followed a normal distribution and the Wilcoxon rank sum test with continuity correction was used for quantitative data. Qualitative data were assessed with chi-squared test.

The results of the two groups after treatment regarding VAS, Levine’s questionnaire, clinical examination and electrophysiological data were then compared using the *t* test. The results in each group before and after treatment were compared using the test for paired data. The differences in numerical data before and after treatment were compared using the *t* test.

## Results

The demographic (Table 1), clinical and electrophysiological data of the two groups showed no statistical differences before treatment (Table 2a). The mean values and standard deviation for age, weight and BMI in both groups were 51.76 (±11.12), 72.76 (±9.54) and 28.19 (±3.24) for group *N* and 53.32 (±13.43), 72.43 (±13.03) and 28.85 (±4.84) for group *P* (Table 1). No complications due to the medication were recorded in either group. After treatment, there were no differences in either group compared to the initial status regarding Durkan’s test, Phalen’s test, VAS, and electrophysiological data (Table 2b). The differences in the most important measurements for the evaluation of CTS before and after treatment are shown in Table 3. For example, the VAS score diminished 0.5 points in group *N* and 0.8 points in group *P*. None of these measurements show a statistically significant improvement in either group. Levine’s questionnaire showed better results in both groups (Table 4) with a statistically significant improvement in only the SSS for group *P* (*p* value of 0.002809) and improvement in the FSS (*p* value of 0.03334) and SSS (*p* value of 0.005) for group *N*. This improvement was not statistically significant between the two groups (Table 2b).

**Table 1:** Average values of demographic data of both groups (SD in brackets)

	Group *N*	Group *P*	*p* value
Age	51.76 (11.12)	53.32 (13.43)	0.6248
Weight	72.76 (9.54)	72.43 (13.03)	0.9104
BMI	28.19 (3.24)	28.85 (4.84)	0.5345

**Table 2:** Average data (a) before treatment in both groups (SD in brackets), (b) after treatment in both groups (SD in brackets)

	Group *N*	Group *P*	*p* value
(a)			
Positive Phalen’s test	4	9	0.1344 (*χ* = 2.2406)
Positive Durkan’s test	17	18	0.9121 (*χ* = 0.0121)
VAS	4.26 (3.41)	4.06 (3.42)	0.8182 (*t* = 0.2309)
FSS	2.03 (0.75)	2.24 (0.66)	0.2469 (*t* = −1.1696)
SSS	2.59 (0.76)	2.57 (0.74)	0.9217 (*t* = 0.0988)
Sensitive latency	3.41 (0.99)	3.32 (1.01)	0.7328 (*t* = 0.343)
Sensitive peak amplitude	10.82 (10.39)	8.86 (5.42)	0.3671 (*t* = 0.9091)
Sensitive speed	40.15(8.08)	40.53 (7.05)	0.8479 (*t* = 0.1927)
Motor latency	4.01 (0.96)	3.87 (0.91)	0.5936 (*t* = 0.5372)
Motor peak amplitude	7.87 (3.7)	7.98 (3.06)	0.9148 (*t* = −0.1076)
(b)			
Positive Phalen’s test	9	13	0.3812 (*χ* = 0.7667)
Positive Durkan’s test	21	19	0.4741 (*χ* = 0.5123)
VAS	3.76 (3.19)	3.25 (3.18)	0.5356 (*t* = 0.6231)
FSS	1.80 (0.81)	1.96 (0.77)	0.4329 (*t* = −0.7896)
SSS	2.25 (0.86)	2.11 (0.81)	0.5078 (*t* = 0.6663)
Sensitive latency	3.33 (0.81)	3.55 (1.6)	0.5264 (*t* = −0.638)
Sensitive peak amplitude	10.93 (7.65)	11.12 (6.13)	0.9208 (*t* = −0.1)
Sensitive speed	38.39 (7.87)	42.00 (8.54)	0.128 (*t* = −1.5489)
Motor latency	3.82 (0.63)	3.71 (0.7)	0.5867 (*t* = 0.5478)
Motor peak amplitude	8.45 (2.74)	8.23 (2.43)	0.7922 (*t* = 0.2652)

**Table 3:** Differences in parameters before and after treatment (SD in brackets)

	*N*	*P*	*p* value
VAS	−0.5 (2.8)	−0.8 (3.08)	0.6867 (*t* = 0.40527)
FSS	−0.22 (0.55)	−0.27 (0.87)	0.7831 (*t* = 0.2765)
SSS	−0.33 (0.6)	−0.45 (0.78)	0.4889 (*t* = 0.69646)
Sensitive latency	−0.1346 (1.4)	0.4026 (1.27)	0.1551 (*t* = −1.4435)
Sensitive peak amplitude	−0.065 (13.28)	1.198 (5.22)	0.6601 (*t* = 0.44248)
Sensitive speed	−1.21 (7.49)	2.13 (9.08)	0.1707 (*t* = −1.3918)
Motor latency	−0.124 (0.44)	−0.057 (0.41)	0.6366 (*t* = 0.47649)
Motor peak amplitude	−0.2413 (2.94)	0.0368 (2.89)	0.7887 (*t* = −0.2702)

**Table 4:** Comparison of pre-treatment and post-treatment changes in each group (SD in brackets)

	Pre-treatment	Post-treatment	*p* value
FSS group *N*	2.03 (0.75)	1.80 (0.81)	0.03334*
FSS group *P*	2.24 (0.66)	1.96 (0.77)	0.08656
SSS group *N*	2.59 (0.76)	2.25 (0.86)	0.005*
SSS group *P*	2.57 (0.74)	2.11 (0.81)	0.002809*

Statistically significative data are indicated

## Discussion

Nerve pressure induces inflammation of nerves and nerve roots, neuritis and radiculitis. Subsequently, they progress into a more chronic pathological state due to the induction of a number of cascades of chemical inflammatory reactions [21]. Inflammatory cells, such as activated mast cells, play an important role in nerve compression syndromes and are one of the sources of pro-inflammatory prostaglandins and cytokines [22].

Neuropathic pain is challenging to manage and different conservative treatments have been proposed in patients with mild to moderate CTS, such as the use of splinting the wrist, ultrasonic therapy, laser therapy, oral steroids, non-steroidal anti-inflammatory drugs, oral vitamin B6, local injection of corticosteroids and work place modifications. Clinical studies have confirmed that a significant short-term benefit could be gained with some of these treatments [23–27]. Although a steroid injection into the wrist is often successful, a major complication such as iatrogenic injury to the median nerve is possible [28].

Some clinical studies suggested an anti-edema and analgesic effect of PEA [7, 29, 30] that could be useful in the conservative treatment of mild and moderate CTS with a low risk of complications. PEA is a safe medication that can be used in the treatment of different compression neuropathies [31] as well as neuropathic pain due to diabetes or trauma [32].

PEA has been used effectively for low-grade CTS, together with other treatment measures. While a daily dose of 600 mg has been effective in other compression neuropathies, our work with this dose in CTS has proved to be insufficient. However, a daily dose of 1200 mg for two months has been proved to be effective in the treatment of CTS associated with diabetes [33]. This is in contrast to work by Conigliaro et al. [1], who reported a clinical improvement and a reduction of median nerve latency time in ENG after treatment with both 600 and 1200 mg of PEA for one month in moderate CTS. This study included a control group but was not blinded, since this group received no treatment. In addition, this study included only 26 patients in total.

There is no electrodiagnostic test that can be considered gold standard. Even a combination of clinical and electrodiagnostic tests does not have sufficient evidence of providing the best performance for CTS diagnosis and grading [34]. However, sensory nerve conduction to the median nerve with distal latency and median motor nerve conduction used in our study are measures that are recommended for electrodiagnostic testing [35].

Our study was designed as a prospective double-blinded randomized trial which would avoid the placebo effect of taking a medication, i.e., the bias of the patient considering he had had an improvement just because he was taking a treatment. Another strong point of our study is that the number of patients is much higher than all of the other studies we have cited. This makes us consider our clinical data to be very reliable. These data indicate a slight clinical improvement in the questionnaire results that needs to be studied further using higher doses of PEA. In contrast, electrodiagnostic testing could introduce some doubts into the results when compared to the results of other published studies; we did not find any changes but other authors have done so, albeit with a small number of patients.

The treatment of mild to moderate CTS includes several therapies that can be combined. A safe medication for a limited time appears to be an appealing option in the treatment of CTS; however, PEA does not seem to fulfill these requirements at the moment. It clearly seems to be a safe medication but its dosage and real effect need further study.

## References

[1] Conigliaro R, Drago V, Foster PS, Schievano C, Marzo VDI (2011). Use of palmitoylethanolamide in the entrapment neuropathy of the median in the wrist. Minerva Med.

[2] Chang C-W, Wang Y-C, Chang K-F (2008). A practical electrophysiological guide for non-surgical and surgical treatment of carpal tunnel syndrome. J Hand Surg Eur.

[3] Keppel Hesselink JM, Kopsky DJ (2015). Palmitoylethanolamide, a neutraceutical, in nerve compression syndromes: efficacy and safety in sciatic pain and carpal tunnel syndrome. J Pain Res.

[4] Mazzari S, Canella R, Petrelli L, Marcolongo G, Leon A (1996). *N*-(2-hydroxyethyl) hexadecanamide is orally active in reducing edema formation and inflammatory hyperalgesia by down-modulating mast cell activation. Eur J Pharmacol.

[5] Costa B, Conti S, Giagnoni G, Colleoni M (2002). Therapeutic effect of the endogenous fatty acid amide, palmitoylethanolamide, in rat acute inflammation: inhibition of nitric oxide and cyclo-oxygenase systems. Br J Pharmacol.

[6] Re G, Barbero R, Miolo A, Di Marzo V (2007). Palmitoylethanolamide, endocannabinoids and related cannabimimetic compounds in protection against tissue inflammation and pain: potential use in companion animals. Vet J.

[7] Costa B, Comelli F, Bettoni I, Colleoni M, Giagnoni G (2008). The endogenous fatty acid amide, palmitoylethanolamide, has anti-allodynic and anti-hyperalgesic effects in a murine model of neuropathic pain: involvement of CB(1), TRPV1 and PPARgamma receptors and neurotrophic factors. Pain.

[8] Jaggar SI, Hasnie FS, Sellaturay S, Rice AS (1998). The anti-hyperalgesic actions of the cannabinoid anandamide and the putative CB2 receptor agonist palmitoylethanolamide in visceral and somatic inflammatory pain. Pain.

[9] Calignano A, La Rana G, Giuffrida A, Piomelli D (1998). Control of pain initiation by endogenous cannabinoids. Nature.

[10] Hesselink K, Hekker J (2012). Therapeutic utility of palmitoylethanolamide in the treatment of neuropathic pain associated with various pathological conditions: a case series. J Pain Res.

[11] Lombardi G, Miglio G, Varsaldi F, Minassi A, Appendino G (2007). Oxyhomologation of the amide bond potentiates neuroprotective effects of the endolipid *N*-palmitoylethanolamine. J Pharmacol Exp Ther.

[12] Sheerin AH, Zhang X, Saucier DM, Corcoran ME (2004). Selective antiepileptic effects of *N*-palmitoylethanolamide, a putative endocannabinoid. Epilepsia.

[13] Keppel Hesselink JM (2011). Glia as a new target for neuropathi pain, clinical proof of concept for palmitoylethanolamide, a glia modulator. Anesth Pain Intensive Care.

[14] Levine DW, Simmons BP, Koris MJ, Daltroy LH, Hohl GG, Fossel AH, Katz JN (1993). A self-administered questionnaire for the assessment of severity of symptoms and functional status in carpal tunnel syndrome. J Bone Joint Surg Am.

[15] Rosales RS, Díez de la Lastra I, Benseny-Delgado E (2002). The evaluation of the Spanish version of the DASH and Carpal tunnel syndrome health related quality of life instruments. Process of adaptation and reliability. J Hand Surg.

[16] Sambandam SN, Priyanka P, Gul A, Ilango B (2008). Critical analysis of outcome measures used in the assessment of carpal tunnel syndrome. Int Orthop.

[17] AAOS (2008) Clinical practice guideline on the treatment of Carpal tunnel syndrome. http://www.aaos.org/Research/guidelines/CTSTreatmentGuideline.pdf. Downloaded on 4-17-2016

[18] Durkan JA (1991). A new diagnostic test for carpal tunnel syndrome. J Bone Joint Surg Am.

[19] Phalen GS (1966). The carpal-tunnel syndrome. Seventeen years’ experience in diagnosis and treatment of six hundred fifty-four hands. J Bone Joint Surg Am.

[20] R_Development_Core_Team (2011). R: a language and environment for statistical computing.

[21] Mulleman D, Mammou S, Griffoul I, Watier H, Goupille P (2006). Pathophysiology of disk-related sciatica. I.-Evidence supporting a chemical component. Joint Bone Spine.

[22] Zuo Y, Perkins NM, Tracey DJ, Geczy CL (2003). Inflammation and hyperalgesia induced by nerve injury in the rat: a key role of mast cells. Pain.

[23] Aroori S, Spence RA (2008). Carpal tunnel syndrome. Ulster Med J.

[24] Barbosa RI, Fonseca MC, Rodrigues EK, Tamanini G, Marcolino AM, Mazzer N, Guirro RR, MacDermid J (2016). Efficacy of low-level laser therapy associated to orthoses for patients with carpal tunnel syndrome: a randomized single-blinded controlled trial. J Back Musculoskelet Rehabil.

[25] Golriz B, Ahmadi Bani M, Arazpour M, Bahramizadeh M, Curran S, Madani SP, Hutchins SW (2016). Comparison of the efficacy of a neutral wrist splint and a wrist splint incorporating a lumbrical unit for the treatment of patients with carpal tunnel syndrome. Prosthet Orthot Int..

[26] Halac G, Demir S, Yucel H, Niftaliyev E, Kocaman G, Duruyen H, Kendirli T, Asil T (2015). Splinting is effective for night-only symptomatic carpal tunnel syndrome patients. J Phys Ther Sci.

[27] Talebi M, Andalib S, Bakhti S, Ayromlou H, Aghili A, Talebi A (2013). Effect of vitamin b6 on clinical symptoms and electrodiagnostic results of patients with carpal tunnel syndrome. Adv Pharm Bull.

[28] Kim HJ, Park SH (2014). Median nerve injuries caused by carpal tunnel injections. Korean J Pain.

[29] Aliamides Jack DB (1996). A new approach to the treatment of inflammation. Drug News Perspect.

[30] Genovese T, Esposito E, Mazzon E, Di Paola R, Meli R, Bramanti P, Piomelli D, Calignano A, Cuzzocrea S (2008). Effects of palmitoylethanolamide on signaling pathways implicated in the development of spinal cord injury. J Pharmacol Exp Ther.

[31] Guida G, de Fabiani A, Lanaia F, Alexandre A, Vassallo GM, Cantieri L, de Martino M, Rogai M, Petrosino S (2010). La palmitoiletanolamida (Normast^®^) en el dolor neuropático crónico por lumbociatalgia de tipo compresivo: estudio clínico multicéntrico. Dolor.

[32] Cocito D, Peci E, Ciaramitaro P, Merola A, Lopiano L (2014). Clinical study short-term efficacy of ultramicronized palmitoylethanolamide in peripheral neuropathic pain.

[33] Assini A, Laricchia D, Pizzo R, Pandolfini L, Belletti M, Colucci M, Ratto S (2010). The carpal tunnel syndrome in diabetes: clinical and electrophysiological improvement after treatment with palmitoylethanolamide. Eur J Neurol.

[34] AAOS (2007) Clinical practice guideline on the diagnosis of Carpal tunnel syndrome. http://www.aaos.org/Research/guidelines/CTS_guideline.pdf. Downloaded on 4-17-2016

[35] AAEM (2002) Practice topic in electrodiagnostic medicine. https://www.aanem.org/getmedia/95a3a91e-a196-4d1c-bbc9-38d4f487faae/CTS.pdf. Downloaded on 4-17-2016

